# A modified tubularised incised plate urethroplasty technique and a revised hypospadias algorithm

**DOI:** 10.4103/0970-0358.63946

**Published:** 2010

**Authors:** Sameek Bhattacharya

**Affiliations:** Department of Plastic Surgery, PGIMER & Dr. RML Hospital Hospital, New Delhi, India

**Keywords:** Algorithm, chordee, hypospadias, snodgrass, urethroplasty

## Abstract

To simplify and standardize surgical management of hypospadias, a modified tubularised incised plate (TIP) urethroplasty (Snodgrass) technique has been described and a revised hypospadias management algorithm has been formulated. The study aims to evaluate the viability of the described procedure in different types of hypospadias and tests the validity of the algorithm. The modification described is recruitment of penile and glandular skin lateral to the urethral plate to facilitate tubularisation. The algorithm starts with penile degloving with preservation of urethral plate. Snodgrass repair was done in cases with no chordee and where skin chordee resolved by skin take down. Modified Snodgrass repair was done in cases where urethral plate was narrow. Another modification proposed by us is single layer penile skin closure instead of an added dartos flap, which was done in both classical and modified Snodgrass repair. Cases of severe chordee not resolved by skin take down were repaired by transverse preputial island flap (TPIF) and Bracka's technique. Dorsal plication was not used as an orthoplasty modality. It was possible to repair 68.89% of the cases by Snodgrass repair. These patients either had no chordee or had superficial skin tethering (skin chordee) which resolved on degolving. All these cases were coronal, distal and mid penile hypospadias. Remaining cases were mid, proximal and penoscrotal with true fibrous chordee and were repaired by TPIF or Bracka's technique. The Snodgrass technique had a fistula rate of 9.67%. Acceptably, low fistula rate and simple execution make the proposed modification of classical Snodgrass repair a viable option. The proposed algorithm proves to be a useful tool for standardised and logical preoperative decision making. It also defines indications of the three techniques vis-à-vis the type of hypospadias.

## INTRODUCTION

The surgical end point of any hypospadias repair is to achieve a normal looking phallus which functions normally. But the infinite number of operative procedures described for this malady reflects the fact that the quest for the "normal phallus" is far from over. Moreover, hypospadias as a clinical entity encompasses a wide array of presentations in terms of meatal position and ventral curvature. Hence, choosing the right procedure for a particular type of hypospadias from this elaborate menu is often a herculean task.

One of the major controversies of hypospadias repair revolves around chordee. The chordee has been an enigma, owing to its varied presentation and its divergent understanding among experts. But recently, with the development of urethroplasty techniques with urethral plate, there is a significant clarity in the understanding of chordee. With this background knowledge of urethroplasty by urethral plate and chordee, the author proposes modified tubularised incised plate (TIP) urethroplasty (Snodgrass repair) and an algorithm to simplify and standardize the repair procedure vis-à-vis the type of hypospadias.

## MATERIAL AND METHODS

**Figure 1:**
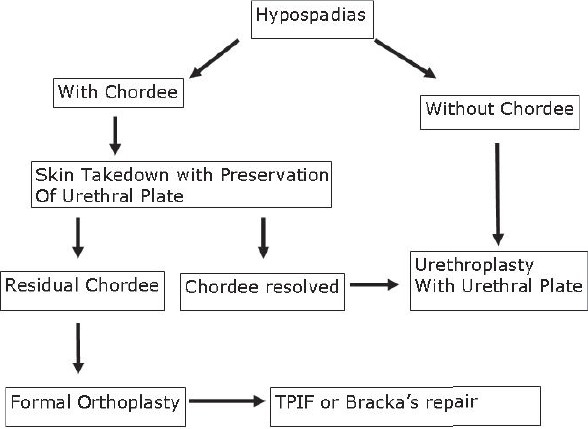
Revised hypospadias algorithm

**Figure 2:**
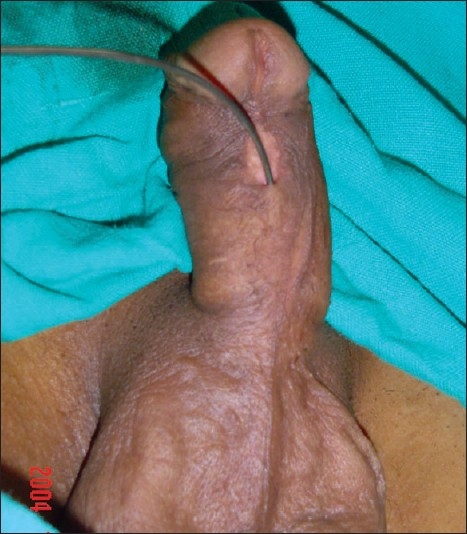
18 years old boy with midpenile hypospadias. The urethral plate is narrow and cannot be tubularised after incision over a 12 F catheter

**Figure 3:**
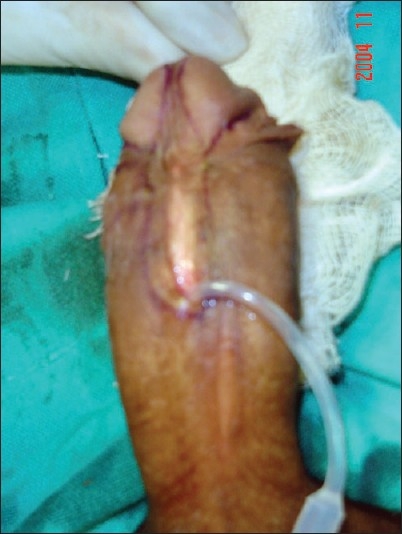
Marking of the incision. Note that adjacent shaft and glandular skin has been included with the urethral plate

**Figure 4:**
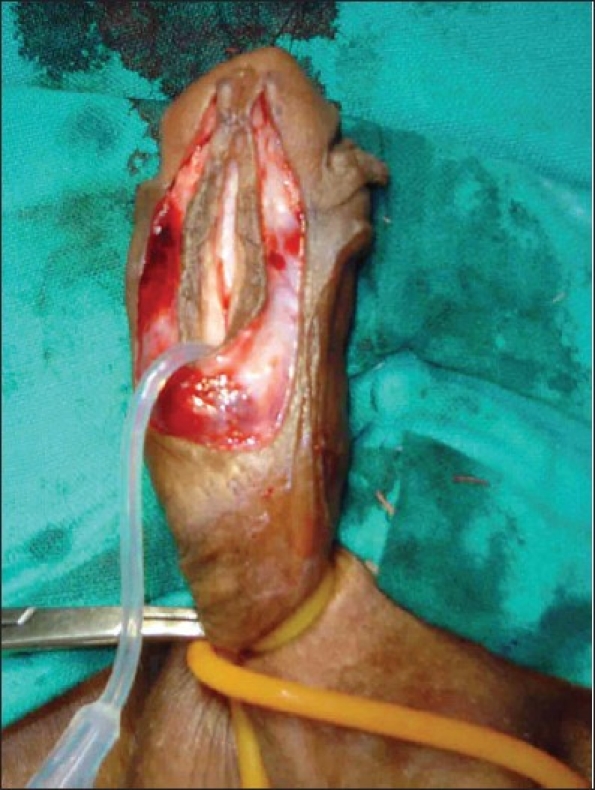
Preserved urethral plate incised in midline. Note that shaft and glandular skin has been taken with urethral plate

**Figure 5:**
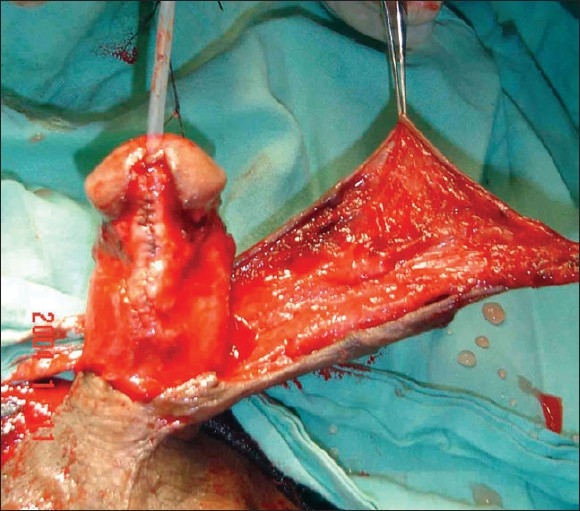
Urethral plate tubularised over 12 F silicon catherter. Urethroplasty done in single layer by continuous subcuticular inverting suture. The suturing is done with 6-0 Polyglycolic acid suture. Penile shaft skin degloved till penoscrotal junction. The skin flap is ready to cover the urethroplsty suture line

**Figure 6:**
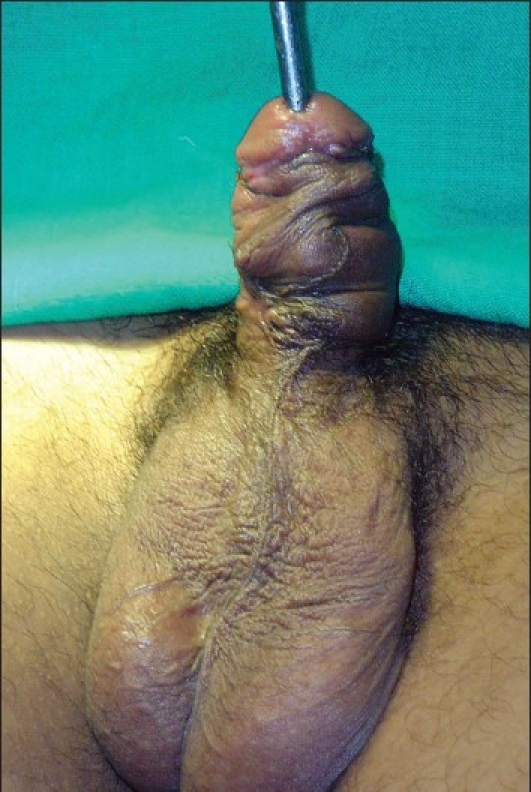
Six months post-op: Note the skin closure line is away from the urethroplasty suture line

**Figure 7:**
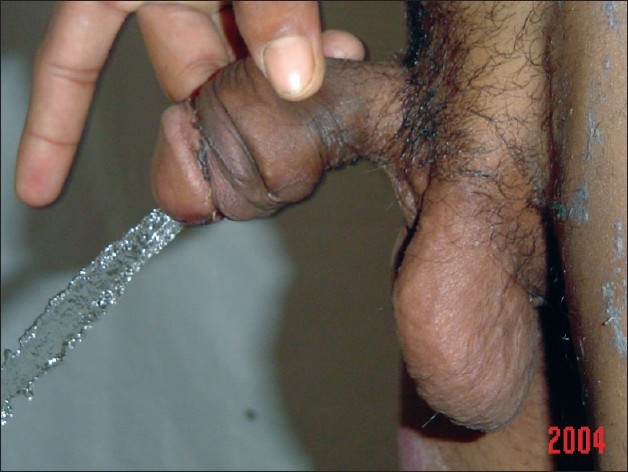
One-year post-op: micturation with good caliber stream

**Figure 8:**
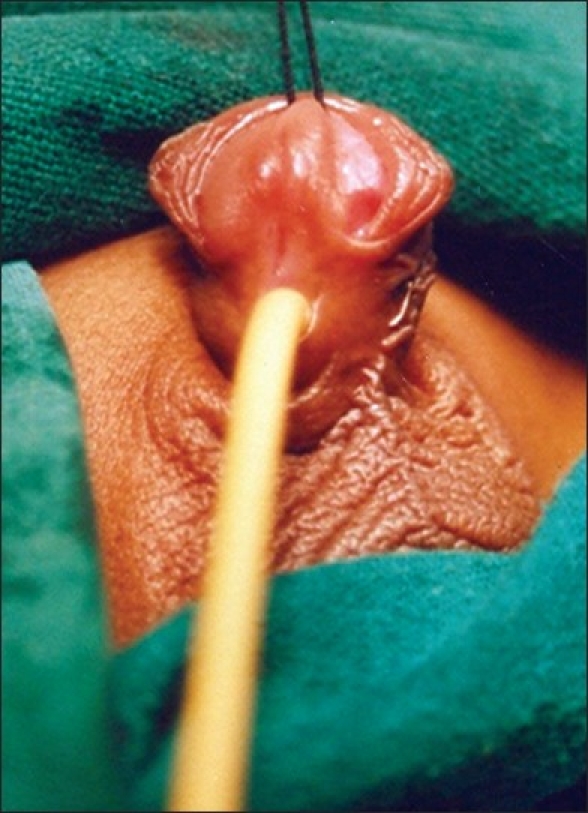
Three-year-old child with distal penile hypospadias with wide urethral plate and spatulated glans. This obviated the need of recruiting skinlateral to the plate

**Figure 9:**
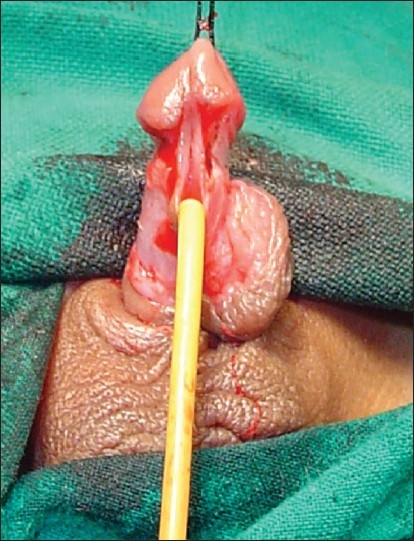
Urethroplasty was done only by urethral plate as in classical Snodgrass repair. Note the preserved urethral plate with longitudinal incision on it. The penis has been degloved till the penoscrotal junction and the shaft skin fl ap is ready for cover

**Figure 10:**
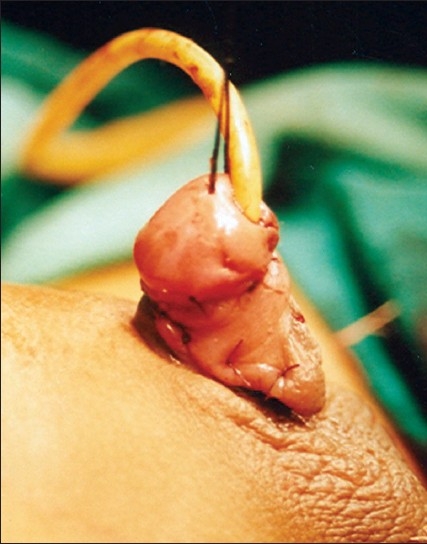
The shaft skin has transposed ventrally. Note the skin closure is away from the urethroplasty towards the dorsum on the right side

**Figure 11:**
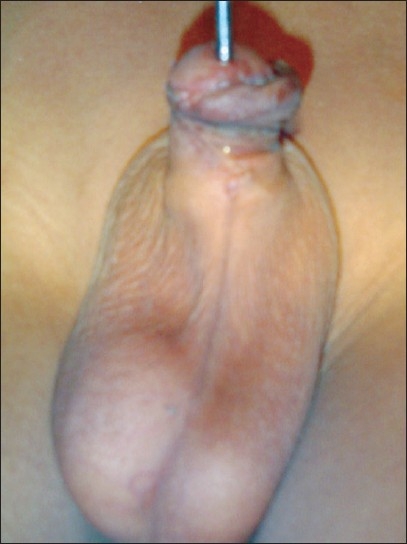
Six months post-op results. Note the neomeatus at the tip of the glans

The study revolves around the fact that not all hypospadias have chordee; however, if chordee is present, it can be due to superficial skin tethering, fibrous chordee or corporal disproportion. The presence of chordee and its exact type can only be determined preoperatively after degloving the penis. The urethroplasty technique depends upon the presence and type of chordee. The algorithm was devised to guide the surgeon in a step-wise manner, first to define the type of chordee and then advise on the technique of repair [Figure 1]. The cornerstone of the algorithm is shaft skin take down with preservation of urethral plate. The degloving of penis commenced with a circumcoronal incision, which extended proximally encircling the urethral plate and preserving it. The degloving was done till the penoscrotal junction. All ventral tethering lateral to the urethral plate was excised. Subsequently, an artificial erection test was done. In case where no chordee was evident, signifying only superficial skin tethering, the next step was urethroplasty by incising and tubularising the urethral plate. We followed the classical TIP urethroplasty techinique described by Snodgrass in most of the cases. But in patients with narrow urethral plate we have devised and used a modification of the classical procedure (Snodgrass Repair). In these cases with narrow urethral plate we recruited little bit of shaft and glanular skin lateral to the plate and then tubularised it after incising it longitudinally [Figures 2 and 3]. In all classical and modified Snodgrass operations, no dartos flap was used during closure; instead the whole degloved skin was transposed ventrally and skin sutured towards dorsum. This way the skin closure was away from the urethroplasty suture line and there was no overlap of suture lines [Figures 4 and 5]. Suturing was single layer in both urethroplasty and skin closure [Figures 6 and 7]. For urinary diversion, Foley's catheter was used. For children less than 5 years, 8 to 10 F catheter and for patients more than 5 years, 12 to 14 F catheters were used. Only silicon catheters were preferred; although in a few cases latex catheter was used as the former was not available. In cases where urethral plate was adequate, classical TIP urethroplasty was performed though the closure was in single layer and suture lines were staggered as described earlier [Figures 8‐11]

If there was chordee after skin take down, a formal orthoplasty was done by excising all ventral tethering including the urethral plate. An artificial erection test was repeated and if no chordee was evident, the next step was urethroplasty or resurfacing. Dorsal plication was not included as an orthoplasty modality. In case the phallus was uncircumcised, urethroplasty was done by a Transverse Preputial Island Flap (TPIF). In a circumcised penis ventral resurfacing was done by split skin graft and urethroplasty was done in a second stage as in Bracka's technique. This algorithm was applied to 45 consecutive primary hypospadias operations. The age of the patients ranged from 2 to 23 years. The dressing protocol was similar in all cases. The phallus was dressed with paraffin gauze, glycerine acriflavin cotton and fluffy gauze followed by compressive bandaging and fixation with elastoplast. The dressing was removed on day 6 and catheter was taken out on day 8. While removing the catheter, the syringe was kept attached to the catheter with the plunger pulled so that the balloon remained collapsed due to negative pressure. This manoeuvre ensured smooth extrusion of the catheter without damaging the suture line.

This was a pilot study to validate and check the feasibility of this algorithm. In the course of this study we also aimed to determine the frequency of skin and fibrous chordee, evaluate the applicability of Snodgrass repair in different types of hypospadias, and define the indications of orthoplasty and other urethroplasty techniques. In order to keep the operating technique uniform, all the procedures were performed by the author only.

## RESULT

Out of 45 patients with hypospadias, 10 were coronal, 14 distal penile, 12 mid penile, 5 proximal penile and 4 penoscrotal. None of the coronal cases and 8 out of 14 distal penile cases had chordee. Remaining 6 distal penile cases had superficial skin tethering. All the mid penile hypospadias had chordee but only 5 had fibrous chordee and rest 7 had only skin involvement. All the proximal penile and penoscrotal cases had fibrous chordee. Hence, 40% (18 out of 45) had no chordee and 28.89% (13 of 45) had only superficial skin tethering which was corrected by skin take down [Table 1]. In these 31 cases (68.89%), urethral plate was preserved and they were managed by Snodgrass repair. Snodgrass repair was possible in all cases of coronal and distal penile hypospadias and 58.33% (6 out of 8) of mid penile hypospadias. Of these 31 cases, 10 (33.33%) patients had a narrow urethral plate and were repaired by the modified Snodgrass technique by recruiting shaft and glandular skin lateral to the urethral plate. We encountered narrow urethral plate in all types of hypospadias. But the need for the modified technique was mainly in adolescent and adult cases where a broad plate is required in order to tubularise over a 12 or 14F catheter. The modified technique was done in 8 patients who were >15 years and only in 2 cases <15 years. One patient of <5 years and 1 patient of 5-15 years needed the modification. Both these cases were mid penile hypospadias. Among the 8 patients of >15 years requiring modified Snodgrass repair, 3 were coronal, 3 were distal and 2 were mid-penile hypospadias [Table 2]. The ventral skin is generally hairless in adults till about mid-penile level. Since the modification was performed only in distal and mid-penile cases, the recruited skin lateral to the urethral plate was hairless.

**Table 1:** Distribution of type of hypospadias, presence of chordee and technique of repair

*Type of hypospadias*	*Number of cases*	*Chordee*		
		*None*	*Superficial skin tethering*	*Fibrouschordee*
Coronal	10	10	0	0
		Classical snodgrass repair 7		
		Modified snodgrass repair 3		
Distal penile	14	8	6	0
		Classical snodgrass repair 6	Classical snodgrass repair 3	
		Modified snodgrass repair 2	Modified snodgrass repair 3	
Mid-penile	12	0	7	5
			Classical snodgrass repair 5	TPIF repair 3
			Modified snodgrass repair 2	Bracka repair 2
Proximal penile	5	0	0	5
				TPIF repair 3
				Bracka repair 2
Penoscrotal	4	0	0	4
				TPIF repair 1
				Bracka repair 3

**Table 2:** Distribution of type of hypospadias, age and technique of repair

*Type of hypospadias*	*Number of cases*	*Age group*		
		*< 5 years*	*5 ‐15 years*	*> 15 years*
Coronal	10	3	3	4
		Classical snodgrass repair 3	Classical snodgrass repair 3	Classical snodgrass repair 1
		Modified Snodgrass Repair 0	Modified snodgrass repair 0	Modified snodgrass repair 3
Distal penile	14	4	6	4
		Classical snodgrass repair 4	Classical snodgrass repair 6	Classical snodgrass repair 1
		Modified snodgrass repair 0	Modified snodgrass repair 0	Modified snodgrass repair 3
Midpenile	12	3	4	5
		Classical snodgrass repair 1	Classical snodgrass repair 1	Classical snodgrass repair 1
		Modified snodgrass repair 1	Modified snodgrass repair 1	Modified snodgrass repair 2
		TPIF repair 1	TPIF repair 1	TPIF repair 1
		Bracka repair 0	Bracka repair 1	Bracka repair 1
Proximal penile	5	3	1	1
		TPIF repair 3	TPIF repair 0	TPIF repair 0
		Bracka repair 0	Bracka repair 1	Bracka repair 1
Penoscrotal	4	1	1	2
		TPIF repair 1	TPIF repair 0	TPIF repair 0
		Bracka repair 0	Bracka repair 1	Bracka repair 2

There were 14 (31.11%) cases with true fibrous chordee, which needed formal orthoplasty. These cases included: 5 mid-penile, 5 proximal penile and 4 penoscrotal types (14 out of 45 cases) had true fibrous chordee. Out of these 14 cases, 7 circumcised cases were repaired by Bracka's repair and 7 uncircumcised cases were repaired by TPIF technique [Table 1]. None of the cases with chordee had corporal disproportion.

Three patients out of 31 operated by Snodgrass repair had fistula (fistula rate of 9.67%). Only 2 patients of TPIF repair had fistula. One of the cases repaired by Bracka had fistula. The patients were followed up for 2 years. Four patients operated by modified Snodgrass repair had meatal stenosis and 3 resolved after regular dilatation for 6 months. One required formal meatotomy.

## DISCUSSION

The lack of standardization of hypospadias repair revolves around varied understanding of chordee and lack of consensus among experts regarding choice of repair. The former contentious issue has been largely put to rest with the advent of urethral plate urethroplasty techniques. The development of this repair followed better clarity in understanding chordee and urethral plate.[1‐4] The major shift in this perception has been distinction between superficial skin tethering and true fibrous contracture as a contributor to chordee.[5,6] Although chordee is present in 25-55% of all hypospadias, a sizable number are peroperatively correctable by penile degloving.[7‐9] Hence, urethral plate urethroplasty emerged as a potent option in majority of cases.[10‐14] However, this knowledge has not paved the way to standardized repair protocol because proponents of different techniques extended specific indications for their respective procedure to in all types of hypospadias.[8,10,15‐19]

There have been attempts in clearing this ambiguity by proposing algorithms.[11,12] The algorithm presented in this study tows this line of standardizing the treatment protocol of hypospadias. In this protocol TIUP repair is indicated in all distal cases with no chordee and in those where chordee is resolved by penile degloving, and is in consonance with other authors.[11,15,20‐22] However, this algorithm deviates from some of the published protocol on two counts. First, dorsal plication is not exercised as an orthoplasty modality in cases with severe chordee where degloving does not suffice and secondly, TIUP is replaced by TPIF and Bracka's[23] repair in proximal cases after formal chordee excision sacrificing urethral plate. We avoid dorsal plication even in older children and adults since it leads to shortening of the penis. The benefit of the algorithm lies in the fact that a surgeon needs to master only three techniques, which he or she can apply objectively in defined indications. Following this system, all types of hypospadias can be managed.

Our study revealed 60% (27 out of 45) had chordee, but true fibrous chordee was present only in 31.11% (14 out of 45) of cases and 13 had superficial skin tethering. Eighteen cases had no chordee. This objective preoperative categorization was feasible by following the algorithm proposed by us. Thirty one patients (68.89%) i.e., 18 without chordee and 13 cases with superficial skin tethering were repaired by Snodgrass repair. This technique could be executed in all coronal and distal hypospadias and 7 out of 12 mid penile hypospadias. The algorithm allowed us to take a preoperative decision in 13 cases of superficial skin tethering which resolved after skin take down allowing Snodgrass repair. None of the proximal and penoscrotal hypospadias allowed modified Snodgrass repair as there was significant chordee after degloving. This is in deviation from number of previous reports, where Snodgrass repair was used in proximal and penoscrotal cases.[8,14‐17] This was possible because these authors used dorsal plication in cases of severe chordee thereby preserving the urethral plate. But the need for judicious use of Snodgrass repair in proximal cases with severe chordee has been proposed by the pioneer of the technique himself.[8] Snodgrass himself recommends staged repair in these cases where a possibility of recurrent curvature and other complications is anticipated.[8] There are even reports of combining the Snodgrass repair with Thiersch-Duplay technique in proximal cases. The former is used to reconstruct the glandular urethra and penile urethroplasty was done with the latter technique.[17]

Out of the 31 cases where urethral plate was utilized, classical Snodgrass repair was possible in 21 cases and rest of the 10 patients had narrow urethral plate necessitating the modification proposed by us. This simple modification of recruiting little bit of shaft and glandular skin lateral to the plate made it possible to tubularize the incised urethral plate. This has extended the reach of this novel technique and obviated the need of more demanding onlay preputial island flap repair.[11,24] Most of the cases (8 out of 10) requiring the modified Snodgrass repair were >15 years of age. These cases needed urethroplasty over a 12 or 14 F catheter. Hence, we feel "narrow" urethral plate was more relative than absolute, depending upon the size of the catheter over which we need to tubularize the urethral plate. We face such situations since a sizable number of our primary hypospadias patients are adolescent and adults.

Our fistula rate with Snodgrass repair and its modification was 9.67% (3 out of 31) which is within the limits of the reported fistula rates by different authors.[8,13‐16,25] A single layer closure without a dartos flap simplifies the technique further without compromising the fistula rate. This is because we transpose the skin ventrally and close the skin on the dorsal aspect, keeping the skin suture away from the urethroplasty. We accept that because of this a median raphe could not be made and led to dog ears. But we feel that this cosmetic shortcoming in face of a simplistic repair with minimal fistula rate, is acceptable. These ventral skin redundancies and dog ear can easily be corrected at a later stage to improve the aesthetics when functional end point has been reached.

## CONCLUSION

The modification proposed further simplifies the classical Snodgrass repair and extends its applicability in older patients with narrow urethral plate. The modifications have not compromised with the benefit of minimal fistula rate of the original technique. The algorithm proposed in the study has paved way for a standardised and logical sequence of preoperative decision making. Till now we did not possess any standard policy of management of hypospadias and specific procedures were chosen on the basis of personal preferences. We have now adopted this algorithm as a policy and all cases of hypospadias are managed in accordance with this concept. The algorithm also defines indications of the three techniques vis-à-vis type of hypospadias. This approach will be beneficial to residents and young surgeons alike.
